# Peatland restoration pathways to mitigate greenhouse gas emissions and retain peat carbon

**DOI:** 10.1007/s10533-023-01103-1

**Published:** 2023-12-08

**Authors:** Ülo Mander, Mikk Espenberg, Lulie Melling, Ain Kull

**Affiliations:** 1 Institute of Ecology and Earth Sciences, University of Tartu, Tartu, Estonia; 2 Sarawak Tropical Peat Research Institute, Kuching, Sarawak Malaysia

**Keywords:** Carbon dioxide, Methane, Nitrous oxide, Paludiculture, Peatland restoration, Rewetting

## Abstract

Peatlands play a crucial role in the global carbon (C) cycle, making their restoration a key strategy for mitigating greenhouse gas (GHG) emissions and retaining C. This study analyses the most common restoration pathways employed in boreal and temperate peatlands, potentially applicable in tropical peat swamp forests. Our analysis focuses on the GHG emissions and C retention potential of the restoration measures. To assess the C stock change in restored (rewetted) peatlands and afforested peatlands with continuous drainage, we adopt a conceptual approach that considers short-term C capture (GHG exchange between the atmosphere and the peatland ecosystem) and long-term C sequestration in peat. The primary criterion of our conceptual model is the capacity of restoration measures to capture C and reduce GHG emissions. Our findings indicate that carbon dioxide (CO_2_) is the most influential part of long-term climate impact of restored peatlands, whereas moderate methane (CH_4_) emissions and low N_2_O fluxes are relatively unimportant. However, lateral losses of dissolved and particulate C in water can account up to a half of the total C stock change. Among the restored peatland types, Sphagnum paludiculture showed the highest CO_2_ capture, followed by shallow lakes and reed/grass paludiculture. Shallow lakeshore vegetation in restored peatlands can reduce CO_2_ emissions and sequester C but still emit CH_4_, particularly during the first 20 years after restoration. Our conceptual modelling approach reveals that over a 300-year period, under stable climate conditions, drained bog forests can lose up to 50% of initial C content. In managed (regularly harvested) and continuously drained peatland forests, C accumulation in biomass and litter input does not compensate C losses from peat. In contrast, rewetted unmanaged peatland forests are turning into a persistent C sink. The modelling results emphasized the importance of long-term C balance analysis which considers soil C accumulation, moving beyond the short-term C cycling between vegetation and the atmosphere.

**Supplementary Information:**

The online version contains supplementary material available at 10.1007/s10533-023-01103-1.

## Introduction

### Importance of peatlands in a changing climate

Peatlands cover only about 3% of the Earth’s terrestrial surface (Gorham 1991) but play a crucial role in the global carbon (C) cycle. They act as significant C stores and are sources or sinks for greenhouse gases (GHG) like carbon dioxide (CO_2_), methane (CH_4_) and nitrous oxide (N_2_O; Frolking et al. 2011). These gases contribute to climate change and are considered crucial anthropogenic GHGs. Due to their high C density, peatlands are globally recognized as vital C reservoirs (Gallego-Sala et al. 2018) accounting for about 21% of the global soil organic C stock, estimated ~ 3000 Pg (Leifeld and Menichetti 2017). Peatlands also serve as substantial stores of organic N, with Northern peatlands alone accumulating 8–15 Pg N. When including tropical peatlands, the global estimations reached up to 26 Pg N (Swenson et al. 2019).

Undisturbed peatlands are currently a C sink (~ 0.1 Pg C y^–1^), a moderate source of CH_4_ (~ 0.03 Pg CH_4_ y^–1^), and a very weak source of N_2_O (~ 0.00002 Pg N_2_O–N y^–1^) (Frolking et al. 2011). However, anthropogenic activities, primarily agriculture and forestry drainage (up to 20% of global peatlands), result in net CO_2_ emissions (~ 0.1 Pg C y^–1^), reduced CH_4_ emissions (10% smaller than in natural conditions), and increased N_2_O emissions (~ 20 times higher than in natural peatlands). Consequently, subsidence and soil degradation contribute nearly 6% of global anthropogenic GHG emissions (Wichtmann et al. 2016). Most likely, the global peatland’s GHG balance has turned to a C source, a slightly smaller CH_4_ source, and a larger (but still small) N_2_O source (Frolking et al. 2011). In Europe, 46% of the remaining peatlands have degraded to the point where peat is no longer actively forming (Swenson et al. 2019).

As typical wetlands, peatlands are severely threatened by drainage, climate change, fires and groundwater extraction (Fluet-Chouinard et al. 2023). However, their restoration is beneficial, enabling C capture and sequestration, and minimizing N_2_O emissions (Leifeld and Menichetti 2017).

The northern hemisphere has experienced the highest warming during winter and early spring (Ljungqvist et al. 2016), leading to more frequent freeze–thaw cycles. Annual precipitation has also increased, particularly during the cold half-year in northern regions (Ljungqvist et al. 2016). Conversely, there are many indications of growing frequency of droughts during the warm season (Chiang et al. 2021). An increase in flash floods is also predicted (Zheng et al. 2022). These changing climatic conditions, droughts, rapid fluctuations in groundwater level (Mander et al. 2021), flash floods (Schindler et al. 2020), and soil moisture conditions (Pärn et al. 2018; Evans et al. 2021; Huang et al. 2021) can create hot spots and hot moments of GHG emission in peatland ecosystems.

### Peatland restoration

Rewetting is a crucial step for conservation or sequestration of C in peatlands previously affected by drainage (Günther et al. 2020). Likewise, rewetting can affect nitrogen cycle and reduce cumulative N_2_O–N emissions by up to 70% in European peatlands (Liu et al. 2022). Usually, peatland restoration and rewetting are considered as synonyms, but in this paper we consider rewetting as increasing water table level compared with previously drained status. In case of restoration we expect that both water table and vegetation are manipulated in the way that enables to achieve ecosystem status similar to pre-drainage. Restoration pathways depend on factors such as initial vegetation of the drained area (Heger et al. 2022; Schaller et al. 2022), nutrient status of the residual peat layer (Kreyling et al. 2021) and the expected time frame for achieving planned ecological and socioeconomic benefits of the restoration.

Rewetting may transform former peat extraction sites into mires, paludicultural land, wet forests or shallow waterbodies. Ecologically, the preferred pathway is to restore them as mires (Wilson et al. 2022), which can be achieved by restoring water level, by a combination of rewetting and the application of peat moss layer transfer technique (Gonzalez-Sargas & Rochefort 2019), or to establish shallow waterbodies in hydrologically complex sites (Christen et al. 2016).

Peatland rewetting for agricultural use (including paludiculture) is challenging but may have a positive short-term effect on CO_2_ capture and GHG mitigation (Maljanen et al. 2010; Wilson et al. 2016a, b) but may not stop long-term peat mineralisation. Rewetting for paludiculture (Wichtmann et al. 2016) usually encompasses *Sphagnum*-based C farming (Gaudig et al. 2017/2018), energy crop cultivation (Hyvönen et al. 2009; Mander et al. 2012; Järveoja et al. 2016a; Kandel et al. 2020); wet forestry (Anadon-Rosell et al. 2022), or cultivation of the reed (Martens et al. 2021) or cattail (De Jong et al. 2021). Berries and other wetland plants are also suitable for paludiculture, but cranberry (*Vaccinium*) is the most suitable pioneer species for mire restoration and long-term C capture (Freeman et al. 2022).

Restoration of drained forests is usually achieved by blocking ditches and restoring water levels (Grand-Clement et al. 2015). Regulating the water regime in restored sites is effective for mitigation of GHG emissions (Järveoja et al. 2016b).

Peatland restoration is vulnerable to hydroclimatic conditions, particularly in the temperate zone. Changing precipitation pattern, increasing temperature and decreasing snowpack are expected to contribute to more frequent extreme events like droughts and torrential rains, resulting in increased vulnerability and interannual variability (Alm et al. 1999; Drollinger et al. 2019). In addition, it is also important to consider the potential substantial losses of dissolved and particulate C from drained and restored peatlands when estimating C budgets (Billett et al. 2010; Rosset et al. 2022).

Due to global warming, northern peatlands are projected to experience increased GHG emissions, particularly during non-growing period (Rafat et al. 2021), while Garcin et al. (2022) highlight a lack of knowledge of hydroclimatic vulnerability of peat C in tropical peatlands. Wet-dry seasonality of GHG fluxes is expected from tropical peatland soils (Inubushi et al. 2003). However, the overall impact of climate change on GHG fluxes needs to be better understood. Current understanding suggests that changes in soil temperature, photosynthesis, and soil moisture drive alterations in net C fluxes (Rafat et al. 2021).

### Restoration versus afforestation of peatlands

Peatland restoration involves rewetting, whereas afforestation of drained peatlands and maintaining their drained condition cannot be considered equivalent to restoration. The key issue lies in the difference in the time scale and the discrepancy in distinguishing between short-term C capture in ecosystem (often observed in studies on GHG exchange between the atmosphere and ecosystem) and long-term C sequestration in soil. When peatlands are drained, C loss from the peat can offset the benefit of long-term CO_2_ sequestration achieved by afforestation (Jurasinski et al. 2020, 2023).

Currently, there is insufficient evidence on the long-term benefits of active afforestation of drained peatlands to mitigate climate change (Jurasinski et al. 2023). Afforestation on drained peatland forests and some former peat extraction areas may provide short-term benefits for climate change mitigation (Mäkiranta et al. 2007; Samariks et al. 2023). However, this approach does not account for the value of long-term C storage in peat. Similarly, intensive forestry on drained peatlands will not restore the peatland ecosystem's flora, fauna, and functions (Haapalehto et al. 2017; Loisel & Gallego-Sala 2022).

In addition, afforested drained peatlands are more susceptible to wildfires (Kohlenberg et al. 2018; Zheng et al. 2023). These risks are exacerbated by more frequent and more intense droughts in the boreal zone (Walker et al. 2019), resulting in losses of burnt wood and substantial C losses from burnt and burning peat layers (Liu et al. 2023). The impacts of severe fires have been devastating drained areas of formerly tropical peatland forests in Southeast Asia (Page et al. 2009).

Restoration-versus-afforestation of peatlands is being debated during the legislation procedure of the European Union Nature Restoration Law (NRL) (Jurasinski et al. 2023).

The general aim of this paper is to assess short-term C capture (GHG exchange between the atmosphere and the peatland ecosystem) and analyse the fluxes in the context of long-term C sequestration in peat of restored (rewetted) peatlands and afforested drained peatlands. As a specific objective we implement a conceptual model that compares changes in the long-term C budget and climate warming mitigation potential in the restored and afforested peatlands.

## Conceptual framework

**Fig. 1:**
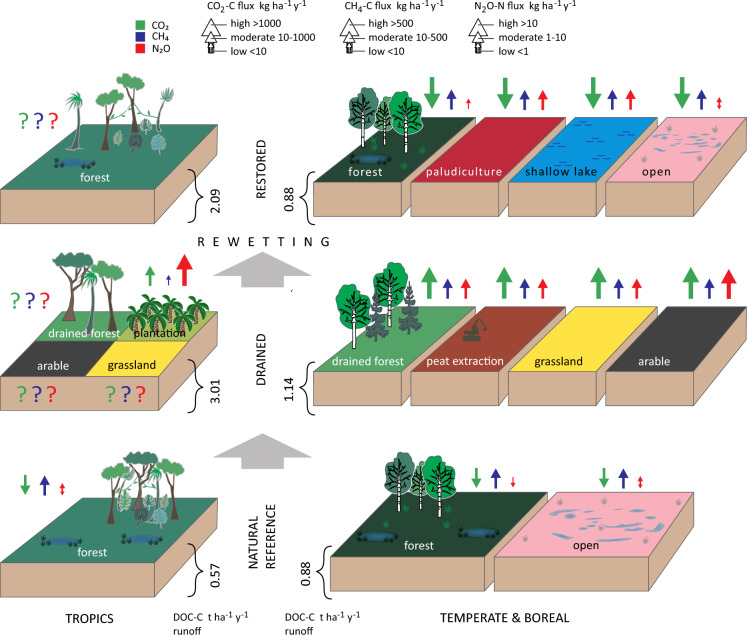
Conceptual figure showing main generalized types of peatland ecosystems from natural and drained to potentially restored peatlands. Proposed set-up for greenhouse gas emission from the main compartments of ecosystems is shown. Arrows indicate literature-based mean values of greenhouse gases CO_2_, CH_4_ and N_2_O fluxes (upward and downward arrows indicate emissions and captures of the corresponding gas, respectively). Question marks indicate scarce data without representative values. Aggregated lateral losses of dissolved organic carbon (DOC) in water are based on Wilson et al. 2016a. Rewetting is displayed as a necessary presumption for peatland restoration. Paludiculture—Sphagnum, berries and energy crops. Open restored areas – bog- and fen-type peatlands

To provide a comprehensive understanding of the framework and to organize the rich source material, we have adopted a three-stage system. These stages refer to peatlands with different water regimes: natural (pristine), drained, and restored (rewetted) ecosystems. Within each stage of water regime, we further divided them into blocks based on primary land use types (Fig. 1). Estimated fluxes of all three GHGs–CO_2_, CH_4_ and N_2_O—are presented using a general three tier scale (high, medium and low fluxes). Flux values are represented by arrows, with upward-pointing arrows indicating emission and downward-pointing arrows indicating capture or uptake of the corresponding gas. In addition, we have indicated the averaged lateral losses of dissolved organic C in water (Fig. 1).

Although our main focus lies on temperate and boreal peatlands, we also briefly discuss tropical peatland forests due to their significance as global hot spots of GHGs, and their large-scale escalating disturbance and destruction. In characterizing GHG fluxes in natural and drained peatlands, we primarily relied on literature sources supplemented by unpublished original data from our research group. However, for tropical wetlands, data is limited or sporadic, resulting in the use of question marks.

**Fig. 2:**
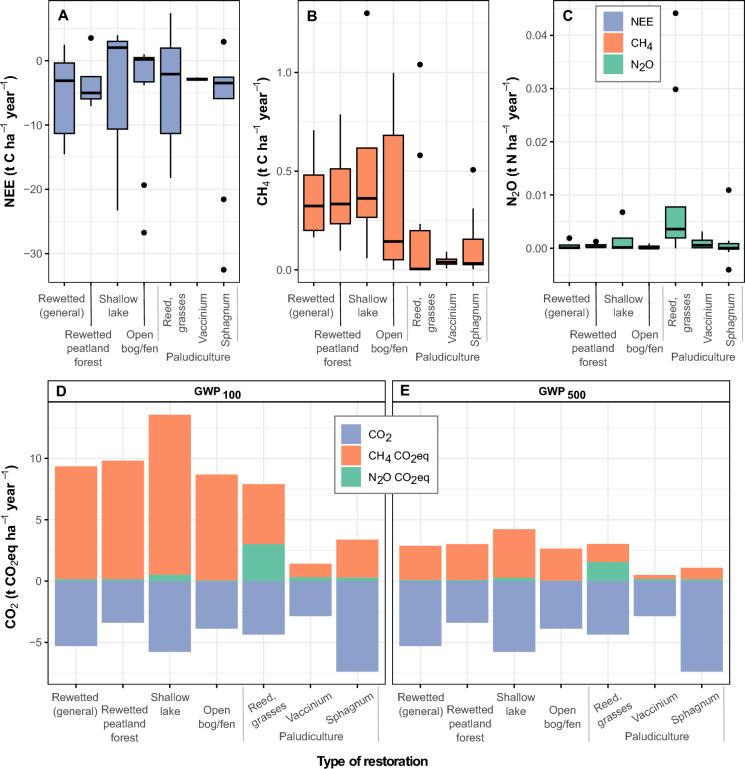
Average annual values of greenhouse gas fluxes in main groups of restored (rewetted) peatlands. **A**–**C** Annual average values of CO_2_-C (NEE), CH_4_-C and N_2_O fluxes. D: greenhouse gas balance in CO_2_equivalent GWP_100_ values (25 for CH_4_, 298 for N_2_O; Myhre et al. 2013). e: greenhouse gas balance in CO_2_equivalent GWP_500_ values (7.6 for CH_4_, 153 for N_2_O; Forster et al. 2007)

For restored (rewetted) peatlands, due to the scarcity and less systematic nature of the data, we present a selection of available GHG flux data in Table 1. The categories of restored (rewetted) peatlands are as in Fig. 1: wet forests, paludiculture, shallow lakes and open mires. Upon no accurate distinction between the mentioned categories in the source data we added a general category – rewetted peatlands – to both Table 1 and Fig. 2. The latter one presents average flux values (Fig. 2A–C) and GHG balances in CO_2_ equivalents (Fig. 2D and Fig. 2E). In the more general Fig. 1, paludiculture is shown as a single category. To compile this information, we referred to both literature sources and results from local research projects in Estonia. For our literature search, we utilized well-known indexing systems such as Web of Science (Clarivate Analytics), Scopus, and Google Scholar. We used following keywords and their combinations: “rewetted peatland(s)”, “restored wetland(s)”, “restored peatland(s)”, “rewetted forest(s)”, “paludiculture”, “Sphagnum farming”, “greenhouse gases, “carbon dioxide “, methane, “nitrous oxide”, “tropical peatlands”. In addition, we looked for references from cited rewetting-related papers.

**Table 1:** Greenhouse gas fluxes in main types of boreal and temperate rewetted peatlands

Type	Name	Coordinates	Country	Period of study	Nutrient status	Plant community/ treatment	NEE (CO_2_-C)	CH_4_-C	N_2_O-N	Source
Rewetted peatlands										
	Twitchell Island old wetland (26 yrs), former agricultural land	38°06’N, 121°38’W	USA, CA	Aug. 2012–Aug. 2013	Rich	*Schoenoplectus acutus, Typha latifolia, T. domingensis, T. angustifolia, Ludwigia* *peploides, Lemna* sp.)	− 3970	387		Knox et al. 2015
	Sherman Island young wetland, 13 yrs), former peatland pasture	38°05’N, 121°42’W	USA, CA	March 2012–Apr. 2013	Rich	*Schoenoplectus acutus*, *Typha* spp.,	− 3680	530		Knox et al. 2015
	Boreal zone aggregated		Global review			All rewetted peatlands (restored from drained forests, grssslands and croplands, and peat extraction sites)	− 1300	123.6	0	Günther et al. 2020
	Temperate zone aggregated		Global review			All rewetted peatlands (see above)	− 400	205.9	0	Günther et al. 2020
	Rewetted organic soils		Germany			Average of both nutrient-poor and nutrient-rich rewetted organic soils	− 0.4	279	0.1	Tiemeyer et al. 2020
	Rewetted peatlands		Global review			Average of 20 literature sources	675	131	1.2	Bianchi et al. 2021
Rewetted peatland forest										
	Rewetted boreal forests		Global review	Before 2016	Poor	Coniferous and mixed peatland forests (soil fluxes) Selected examples from IPCC 2014 Wetlands Supplement	− 1520	73	0.16	Wilson et al. 2016a
					Rich	Coniferous and mixed peatland forests (soil fluxes)	− 1930	220	0.16	Wilson et al. 2016a
	Rewetted temperate forests		Global review	Before 2016	Poor	Coniferous, deciduous and mixed peatland forests (soil fluxes)	− 1220	160	0.16	Wilson et al. 2016a
					Rich		960	418	0.16	Wilson et al. 2016a
	28 rewetted peatland forests		Finland	1 year (2008–2019)	Rich	Fen forests (soil fluxes)			0.8	Minkkinen et al. 2020
					Poor	Bog forests (soil fluxes)			0.5	Minkkinen et al. 2020
	Wöpkendorf *Alnus glutinosa*	54°7′36″N, 12°29′04″E	Germany	May 2018–Apr. 2020	Rich	Rewetted peatland alder forest; *Carex acutiformis, C. riparia*, *Hottonia palustres*, *Solanum dulcamara*		15 to 565		Köhn et al. 2021
Paludiculture										
*Reed/grasses*	Lavassaare *Phalaris*	58°34′20″N, 24°23′ 15″E	Estonia	May 2010–May 2011	Rich	*Phalaris arundinacea* fertilized		3.56	− 0,006	Mander et al. 2012
					Rich	*P. arundinacea non-*fertilized		1.75	− 0.03	Mander et al. 2012
				Jan.-De.c 2014	Rich	*P. arundinacea* fertilized	790	0.14	2.39	Järveoja et al. 2016a
					Rich	*P.arundinacea non-*fertilized	2010	0.18	1.08	Järveoja et al. 2016a
	Keressaare *Phalaris*	58°36′56″N, 27°00′ 36″E	Estonia	Apr. 2015–Mar. 2018	Poor	*P.arundinacea* high water table, fertilized, limed	− 892	1.14	19.0	Maddison et al. 2018
						*P.arundinacea* low water table, fertilized, limed	− 249	3.07	28.1	Maddison et al. 2018
	Gersloot *Typha latifolia*	53°01’N, 5°55’E	Nether-lands	Feb–June 2017	Rich	Fen peat, lab experiments		72.9	2.18	Vroom et al. 2018
	Øby *P. arundinacea*, *Poa spp*	56°27′32″N, 9°40′40″E	Denmark	Mar. 2015–Mar.2017	Rich	*P.arundinacea* flooded plots	− 3970 to − 5970	450 to 1,110	4.0 to 5.5	Kandel et al. 2020
					Rich	*P.arundinacea* semi-flooded plots	− 2650 to − 4990	160 to 710	4.0 to 6.0	Kandel et al. 2020
	Emergent crops		Global review			Average of 6 literature sources		174	1.68	Bianchi et al. 2021
*Vaccinium spp.*	Saint-Louis-de-Blandford,	46°15’N, 72°00’W	Canada, Quebec	Growing season 2012, 2013	Poor	Cultivated cranberry (Vaccinium macrocarpon)		27	0.61	Lloyd et al. 2019
	Maima	58°35′54″N, 24°22′ 36″E	Estonia	(a)	Poor	Naturally regenerated cranberry (*Oxycoccus palustris*)	− 799	68.5	2.0	Burdun et al. 2021
	Laiuse	58°47′17″N, 26°31′ 47″E	Estonia	(a)	Poor	Naturally regen. Cranberry (*Oxycoccus palustris*)	− 837	31.4	− 0.002	Viru et al. 2020; Burdun et al. 2021
	Ess-soo	57°54′51″N, 26°41′ 26″E	Estonia	(a)	Poor	Naturally regen. cranberry (*Oxycoccus palustris*)	− 707	5.4	0.08	Viru et al. 2020; Burdun et al. 2021
*Sphagnum* spp	Nordhümmlinger Moore *Rewetted former peat mining area* Sphagnum site	53 °02’ N, 7°29’ E	Germany	June 2010–Dec. 2011	Poor	*Sphagnum cuspidatum, Eriophorum angustifolium, Molinia caerulea*	− 949	233	− 2.58	Beyer & Höper 2015
	Sphagnum cultivation			June 2010–Dec. 2011	Poor	*S. papillosum, S. cuspidatum, S. palustre, S. fallax, E. angustifolium, Erica tetralix, Juncus effusus, Betula pendula, Drosera spp.*	− 987	24	0.55	Beyer & Höper 2015
	Hankhauser Moor, rewetted former bog grassland	53°16’ N, 08°18’ E	Germany	Sep. 2011–ug. 2013	Poor	*Sphagnum palustre* cultivation	− 5470 to − 6290	10–14	0.01	Günther et al. 2017
				Sep. 2011–Aug. 2013	Poor	*Sphagnum papillosum* cultivation	− 8750 to − 8980	12–27	− 1.0 to 1.0	Günther et al. 2017
	Ess-soo	57°54′51″N, 26°41′ 26″E	Estonia	(a)	Poor	Dredged hollow with naturally regenerated Sphagnum	− 702	380	0.07	Viru et al. 2020; Burdun et al. 2021
	Maima	58°35′54″N, 24°22′ 36″E	Estonia	(a)	Poor	Dredged hollow with naturally regenerated Sphagnum	− 740	116	− 0.11	Viru et al. 2020; Burdun et al. 2021
	Provinzial-moor	52°40’ N, 07°06’ E	Germany	Mar. 2017–Mar. 2019	Rich	*S. papillosum* *S. palustre*; ditch irrigation	− 600 to 2200	3 to 53	0.5 to 1.3	Oestmann et al. 2022
	Drenth	52°41’ N, 07°05’ E	Germany		Rich	*S. papillosum* drip irrigation	700 to 900	1 to 4	1.9 to 12	Oestmann et al. 2022
	Hankhauser Moor	53°16’ N, 08°18’ E	Germany	2015–2022	Rich	*Sphagnum spp,* production field	− 1,609 ± 739	22.7 ± 6.7	− 0,47 ± 1.57	Daun et al. 2023
Shallow lake										
	Giel’cikau Kasyl	52°38′N, 25°21’E	Belarus	Aug. 2010- Aug. 2012	Rich	*Phragmites australis*, *Lemna spp*	− 4,453 to − 8,242	480 to 1470	0 to 0.005	Minke et al. 2016
	Västkärr	59°06′ N, 14°45′ E	Sweden	2010–2014, snow-free periods (65% yr)	Rich	Graminoids, *Carex* spp, *Typha* spp		44	4.3	Jordan et al. 2020
	Zilakalna, Tevgarsu	57°36’ N, 25°10’ E; 57°40’ N, 24°57’ E	Latvia	Dec. 2016–Dec. 2022	Poor	Permanently flooded peat extraction area; no vegetation	550 ± 5	251 ± 77	0.00 ± 0.06	Bardule et al. 2023
	Laiuse	58°47′17″N, 26°31′ 47″E	Estonia	Sep. 2019–June 2023	Poor	14 ha, open water, *P. australis*, Typha & sedges in littoral	1,079	292	0.17	Burdun et al. 2021
Open bog/fen										
	Bois-des-Bel, restored bog	47°57’N, 69°26’W	Canada, Quebec	May–Oct. 2000, 2001, 2002	Poor	*Sphagnum spp., Polytrichum spp., Chamaedaphne calyculata, Vaccinium angusti-folium, Ledum groenlandicum, E. vaginatum,* *T. latifolia*	− 4,330 to − 10,253			Waddington et al. 2010
	Wandering River restored bog (former horticultural area)	55°11’N, 112°29’W	Canada, Alberta	July–Sep. 2011, May–July 2012	Poor	*Sphagnum spp, Carex spp., E. vaginatum, Salix pedicellaris, Polytrichum strictum, Pohlia nutans*	− 11,647 to 1,095	− 3.23 to 540		Strack et al. 2014
	Trebel River valley Rewetted fen, former agric. grassland Phragmites	54°060’N, 12°44’E	Germany	March 2011–March 2012	Rich	*Phragmites australis*	− 830 to 320	110		Günther et al. 2015
	Typha				Rich	*Carex spp*	− 430 to 710	105		Günther et al. 2015
	Carex				Rich	*Typha spp.*	− 30 to 290	590–630		Günther et al. 2015
	Himmel-moor Rewetted in 1981 Heath site	53°44′20’’N, 9°50′58’’E	Germany	Aug. 2010–Jan. 2012	Poor	*Erica tetralix, Calluna vulgaris, V. oxycoccus, Andromeda polifolia*	84	478	− 0.16	Vanselow-Algan et al. 2015
	Sphagnum			Aug. 2010–Jan. 2012	Poor	*Sphagnum spp*	16	748	0.34	Vanselow-Algan et al. 2015
	Purple moor grass			Aug. 2010–Jan. 2012	Poor	*Molinia caerulea*	67	1.114	0.26	Vanselow-Algan et al. 2015
	Burns Bog Ecol. Cons. Area (BBECA) Restored peat mining area Rewetted Cleared	49°06′37"N 123°00′03"W	Canada, British Columbia	June–Aug. 2014	Poor	*Ledum groenlandicum, Betula pendula* *V. corymbosum Sphagnum capillifolium,*		240	0.0075	Christen et al. 2016
	Rewetted Sedge	49°07′09"N 123°00′01"W	Canada, British Columbia	June–Aug. 2014	Poor	*Rhynchospora alba, Dulichium arundinaceum. Sphagnum spp., L. groenlandicum, V. uliginosum*		669	0.005	Christen et al. 2016
	Bellacorick	54°07′ 30’’ N, 09 °33′ 22´´W	Ireland	Nov2008– Dec 2013	Poor	*Sphagnum spp*, *Juncus effusus* *Eriophorum spp.*	− 1,040 ± 800	90 ± 20	0	Wilson et al. 2016b
	Tässi	58°32′ 16’’ N, 25 °51′ 43´´ E	Estonia	Mar. 2014–Mar. 2015	Poor	Bryophytes, Sphagnum; high water level	50	2.0 ± 0.9	− 0.01 ± 0.02	Järveoja et al. 2016b
					Poor	Bryophytes, Sphagnum*, herbs, shrubs*; low water level	− 247	0.9 ± 0.5	0.2 ± 0.1	Järveoja et al. 2016b
	Seba Beach	53°27′ 17″ N, 114°52′50″W	Canada, Alberta	May–Sep. 2015	Poor	All plots (moss, bare, *Eriophorum)*			− 0.135	Brummell et al. 2017
	Seba Beach	53° 33′N, 114° 44’W	Canada, Alberta	May–Aug. 2016, May–Aug. 2017	Poor	*Sphagnum spp*, sedges (rest. 2009)		21		Bieniada & Strack 2021
					Poor	Graminoids, *Polytrichum* moss, *Sphagnum spp*, sedges (rest. 2012)		703		Bieniada & Strack 2021
	Bois-des-Bel	47°58′2″ N 69°25′43″ W	Canada, Quebec	Nov. 2013–Oct. 2014	Poor	*Sphagnum spp*, sedges	− 900 ± 180	44 ± 2		Nugent et al. 2018
	Uchter Moor Rewetted since 1999 former peat mining area		Germany	Jan–Dec. 2017	Rich	*Sphagnum spp, Eriophorum vaginatum, Molinia caerulea E. angustifolium*	262 ± 33	48.8 ± 9.8	0.57 ± 0.21	Schaller et al. 2022

Negative values correspond to sequestration, positive values indicate emission to the atmosphere. Units: kg C ha^−1^ yr^−1^; kg N ha^−1^ yr^−1^

Knox et al.: g CO_2_–C m^−2^ yr^−1^; g CH_4_–C m^−2^ yr^−1^; eddy covariance (FMA analyser).

Günther et al. 2020: t CO_2_ ha^−1^ yr^−1^; kg CH_4_–C ha^−1^ yr^−1^; kg N_2_O–N ha^−1^ yr^−1^.

Bianchi et al.: t CO_2_-eq ha^−1^ yr^−1^; conversion factor of climate–carbon feedbacks: CH_4_ = 34 and N_2_O = 298 (Myhre et al. 2013).

Tiemeyer et al.: t CO_2_–C ha^−1^ yr^−1^; kg CH_4_–C ha^−1^ yr^−1^; kg N_2_O–N ha^−1^ yr^−1^, annual average.

Wilson et al. 2016a: t CO_2_-eq ha^−1^ yr^−1^; conversion factor of climate–carbon feedbacks: CH_4_ = 34 and N_2_O = 298 (Myhre et al. 2013).

Minkkinen et al.: g N_2_O–N m^−2^ yr^−1^; chambers, GC; average annual values.

Köhn et al.: kg CH_4_ ha^−1^ yr^−1^;chambers, GC, Los Gatos laser analyser.

Mander et al.: mg CO_2_–C m^−2^ h^−1^; μg CH_4_–C m^−2^ h^−1^; μg N_2_O–N m−2 h^−1^; chambers, GC, annual median values.

Järveoja et al. 2016a: g CO_2_–C m^−2^ h^−1^; g CH_4_–C m^−2^ h^−1^; chambers, GC, average annual values.

Maddison et al.: mg CO_2_–C m^−2^ h^−1^; µg CH_4_–C m^−2^ h^−1^; µg N_2_O–N m^−2^ h^−1^; chambers, GC; average annual values.

Vroom et al.: mg CH_4_ m^−2^ h^−1^; mg N_2_O m^−2^ d^−1^; chambers on mesocosms in lab, Picarro G2508.

Kandel et al.: Mg CO_2_–C ha^−1^ yr^−1^; Mg CH_4_–C ha^−1^ h^−1^; mg N_2_O–N m^−2^ h^−1^; chambers, GC; range between average annual values.

Lloyd et al.: μg CO_2_–C m^−2^ h^−1^; μg CH_4_–C m^−2^ h^−1^; μg N_2_O–N m^−2^ h^−1^; chambers, GC, vegetation period average values.

Burdun et al.: mg CO_2_–C m^−2^ h^−1^; µg CH_4_–C m^−2^ h^−1^; chambers, GC, average annual values.

Viru et al.: mg CO_2_–C m^−2^ h^−1^; µg CH_4_–C m^−2^ h^−1^; μg N_2_O–N m^−2^ h^−1^; chambers, GC, average monthly and annual values.

Oestmann et al. 2022: t CO_2_ ha^−1^ yr^−1^; g CH_4_–C m^−2^ yr^−1^; mg N_2_O–N m^−2^ h^−1^; chambers, GC, range between average annual values of sub-sites.

Beyer & Hõper 2015: g CO_2_ C m^−2^ yr^−1^; g CH_4_–C m^−2^ yr^−1^, mg N_2_O–N m^−2^ yr^−1^; chambers, GC, average annual values.

Günther et al. 2017: g CO_2_m^−2^ yr^−1^; g CH_4_ m^−2^ yr^−1^; mg N_2_O m^−2^ yr^−1^; chambers, GC, average annual.

Daun et al.: g CO_2_ m^−2^ yr^−1^; g CH_4_ m^−2^ yr^−1^; g N_2_O m^−2^ yr^−1^; chambers, GC, average annual ± sd.

Minke et al.: mg CO_2_–C m^−2^ h^−1^; µg CH_4_–C m^−2^ h^−1^; µg N_2_O–N m^−2^ h^−1^; chambers, LiC or (CO_2_); GC (CH_4_, N_2_O), average annual values.

Jordan et al.: mmol CH_4_ m^−2^ h^−1^; μmol N_2_O m^−2^ h^−1^; average annual values.

Bardule et al.: mg CO_2_–C m^−2^ h^−1^; mg CH_4_–C m^−2^ h^−1^; μg N_2_O–N m^−2^ h^−1^; chambers, GC, average annual ± se.

Waddington et al.: g CO_2_ m^−2^ d^−1^; chambers, IRGA analyser, growing season average values.

Strack et al.: g CO_2_ m^−2^ d^−1^; mg CH_4_ m^−2^ d^−1^chambers, IRGA analyser, growing season average values.

Günther et al.: g CO_2_ C m^−2^ yr^−1^; g CH_4_–C m^−2^ yr^−1^, chambers, GC, average annual values.

Vanselow-Algan et al.: g CO_2_ m^−2^ yr^−1^; μg CH_4_–C m^−2^ s^−1^; μg N_2_O–N m^−2^ s^−1^, chambers, GC, average annual values.

Christen et al.: g CO_2_m^−2^ d^−1^ with EC; nmol CH_4_ m^−2^ s^−1^ and nmol N_2_O–N m^−2^ s^−1^ with chambers & GC, June–August median values.

Wilson et al. 2016b: mg CO_2_–C m^−2^ h^−1^; mg CH_4_–C m^−2^ h^−1^; μg N_2_O–N m^−2^ h^−1^; chambers, GC, average annual ± sd.

Järveoja et al. 2016b: mg CO_2_–C m^−2^ h^−1^; μg CH_4_–C m^−2^ h^−1^; μg N_2_O–N m^−2^ h^−1^; chambers, GC, average annual values.

Brummell et al.: mg N_2_O-N m^−2^ d^−1^, chambers, GC, average annual values.

Bieniada & Strack: µg CH_4_–C m^−2^ h^−1^, Los Gatos; average annual values, chambers.

Nugent et al.: g CO_2_-C m^−2^ yr^−1^; eddy covariance; mean ± sd.

Schaller et al.: EC LiCor g CO_2_ m^−2^ yr^−1^; EC LosGatos g CH_4_ m^−2^ yr^−1^ and mg N_2_O-N m^−2^ yr^−1^, average annual ± standard error values.

^a^NEE CO_2_ measurements from July 2021 until June 2023: CO_2_ Reco, CH_4_ and N_2_O measurements from August 2017 until June 2023.

**Fig. 3:**
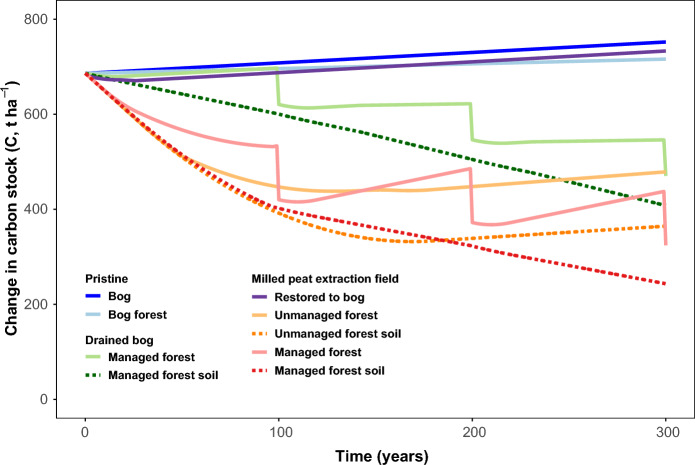
Conceptual figure of C balance in hemiboreal ombrotrophic afforested peatland sites in a 300-year time-span. For drained and restored peatlands, time since drainage is shown. The tabs on the managed peatland forests graphs represent 100-year logging cycles. See Supplementary Table 1 for input parameters and modelling assumptions

To explain the dilemma of peatland restoration and afforestation in a long-term perspective and mitigation of climate warming, we created a conceptual model that helps to characterize the changes in the C budget under different management practices (Fig. 3).

The resulting climatic effects of these management options are strongly time-dependent and rely on difference in forming above or below ground carbon pool and carbon turnover rate.

Our conceptual model of long-term C stock dynamics over 300-year period is based on the approach proposed by Minkkinen & Laine (1998). We developed it further to include C stock dynamics in nutrient-poor mires (open bog and bog forest), drained bog forest, and potential land use scenarios after milled peat extraction (restored bog, naturally regenerated unmanaged forest and managed forest). In modelling, we assumed equal C stock 686 t ha^−1^ in any peatland as initial status, corresponding to C stock with 2 m peat depth in pristine bog (Supplementary Table 1). As approximation, the stem, branches and coarse root biomass were assumed to have stable ratio (branches 12% of stem biomass and coarse roots 19% of above ground biomass) in any age group, wood density (kg m^3^) and C content (%) values were derived from literature (Kask and Pikk 2009; Külla 1997). Initial value of stem biomass was set 3 m^3^ ha^− 1^ for pristine open bog, 100 m^3^ ha^−1^ yr^−1^ for pristine bog forest and 0 m^3^ ha^−1^ for any other land use classes (initially harvested peatland forest or treeless peat extraction site) with annual stem biomass increment by 0 m^3^ ha^−1^ yr^−1^ for open bog, 0.1 m^3^ ha^−1^ yr^−1^ for stable continuous cover bog forest, 4 m^3^ ha^−1^ yr^−1^ for drained peatland forest and naturally regenerated forest on peat extraction site (initial value 0 m^3^ ha^−1^ yr^−1^ until year 20, thereafter gradually increasing until 4 m^3^ ha^−1^ yr^−1^ by year 50 and starting to decline to 0.1 m^3^ ha^−1^ yr^−1^ by year 160 as equal to pristine bog forest) while mean annual increment of 6 m^3^ ha^−1^ yr^−1^ was assigned to managed forest on drained former peat extraction site because of higher fertility of deeper peat layers (usually fen peat) after removal of Sphagnum peat. In pristine bog forest, restored bog and unmanaged peatland forest on former peat extraction site no harvest and timber removal is considered. Harvesting cycle is assumed to be 100 years in both restored managed peatland forest and drained managed peatland forests, while only stem biomass is removed. Fine root biomass was considered as part of C turnover and part of net ecosystem exchange (NEE). NEE values for modelled ecosystems are based on original data from Estonian study sites (Table 1), Estonian national GHG inventory, and literature (Salm et al. 2012; Wilson et al. 2016a). A conceptual scheme presents modelled long-term C stock dynamics in pristine mire ecosystems, restored and afforested drained peatlands (Fig. 3).

## Results and discussion

### Greenhouse gas fluxes in restored peatlands

Figure 1 illustrates a conceptual view of estimated GHG fluxes in natural, drained and restored (rewetted) peatlands. In restored peatlands, CO_2_ fluxes play the main role in climate impacts, while CH_4_ emissions are moderate and N_2_O fluxes are low making them less significant. Lateral losses of dissolved and particulate C in water can account up to a half of total C budget. Among the different restored peatlands, paludiculture has the highest estimated carbon dioxide (CO_2_) sequestration rate (> 1000 kg C ha^−1^ y^−1^) followed by rewetted forests, open fens and bogs. However, some rewetted peatlands are potential source of both CO_2_ and CH_4_, at least during the first 20 years after restoration (Vanselow-Algan et al. 2021).

According to our analysis, all restored peatlands were C sinks. However, there was significant variation of the data, mainly due to limited data availability and differences in the age since rewetting, as well as variation in plant cover development (Table 1). Average annual CO_2_–C stored in rewetted forests, open peatlands and paludicultural sites was − 928, − 534, and − 528 kg CO_2_–C ha^−1^ yr^−1^, respectively (Table 1). These findings are consistent with the CO_2_ flux values reported by Günther et al. (2020) for rewetted peatlands. Due to their diversity, paludicultural ecosystems showed the most significant variation in CO_2_-C fluxes ranging from high C capturing to moderate emissions, with the highest values observed in *Phalaris arundinacea* and *Poa* spp. plantation on rich fen peat (Kandel et al. 2020). Shallow lakes established on flooded peat extraction sites generally emitted low to moderate levels of CO_2_ except extensively vegetated eutrophic sites (Minke et al. 2016).

In all rewetted peatlands, the average annual CH_4_ fluxes were at moderate level, with the highest values observed in shallow lakes (451 kg CH_4_–C ha^−1^ yr^−1^) and followed by rewetted forests, open bogs/fens and paludiculture (218, 163, and 122 kg CH_4_–C ha^−1^ yr^−1^, respectively; Table 1). Average annual N_2_O fluxes ranged from low to moderate levels (0.01 to 4.45 kg N_2_O–N ha^−1^ yr^−1^), except in *Phalaris* paludiculture plantations on poor and acid peat where liming and fertilization resulted in fluxes of up to 19 kg N_2_O–N ha^−1^ yr^−1^ under high groundwater levels and 28.1 kg N_2_O–N ha^−1^ yr^−1^ under low water levels (Maddison et al. 2016; Table 1).

In management of drained peatlands, global warming potential (GWP) of GHG-s should be considered to avoid making decisions based solely on short-period benefits that may overlook the long-term climate cooling effect. Drained peatlands are known to be persistent CO_2_ emitters over the long term, while rewetted peatlands as resilient re-established mire ecosystems effectively contribute to mitigating climate change, even considering radiative forcing of increased CH_4_ emissions (Günther et al. 2020) and decreased N_2_O emissions. Figure 2D and E demonstrate the climate effect of rewetted peatland ecosystems in GWP_100_ and GWP_500_ timeframes. Mires are the only terrestrial ecosystems capable to continuously sequester atmospheric carbon in the long term (Gorham et al. 2012; Cobb et al. 2020), i.e., the carbon sequestration itself is more important than its compound (methane vs carbon dioxide).

For restored (rewetted) peatlands see literature sources in Table 1.

For natural reference and drained peatlands the following literature sources served as the basis of this figure: Abdalla et al. 2016; Aitova et al. 2023; Bardule et al. 2023; Bianchi et al. 2021; Bieniada & Strack 2021; Brummell et al. 2017; Burdun et al. 2021; Busman et al. 2023; Clement et al. 2020; Couwenberg et al. 2010; Daun et al. 2023; Frolking et al. 2011; Griffis et al. 2020; Günther et al. 2020; Hergoualc’h and Verschot 2014; Hyvönen et al. 2009; Inubushi et al. 2003; Järveoja et al. 2016a,b; Jauhiainen et al. 2008, 2012, 2019, 2023; Jordan et al. 2020; Kandel et al. 2020; Kull 2016; Maddison et al. 2016; Mander et al. 2008, 2012, 2016; Melling et al. 2007; Nugent et al. 2018; Oestmann et al. 2022; Oktarita et al. 2017; Pärn et al. 2023; Petrescu et al. 2015; Rosset et al. 2022; Sakata et al. 2015; Salm et al. 2012; Sjögestren et al. 2011; Takakai et al. 2006; Tang et al. 2018; Toma et al. 2011; Truu et al. 2020; Turetsky et al. 2014; Veber et al. 2021, 202X; Wilson et al. 2016a,b; Wong et al. 2018.

### Modelling approach

To estimate the carbon balance of afforested drained and restored rewetted peatlands we developed a conceptual model. Based on the availability of data from our research projects and similar studies in other countries we chose hemiboreal ombrotrophic peatlands as a modelling example. Figure 3 presents estimated long-term dynamics of C stock of afforested drained and restored rewetted hemiboreal ombrotrophic peatlands and their reference pristine bog ecosystem. In a 300-year perspective, considering stable climate conditions, drained forest peat (former bogs) will lose 1.8 and 1.0 t C ha^−1^ yr^−1^, respectively, due to peat mineralization. In afforested peatlands under continuous drainage, C losses from peat reach 0.9 t C ha^−1^ yr^−1^, whereas ecosystem C losses (the peat + vegetation budget and 100-year timber-harvesting regime) are 0.4–0.6 t C ha^−1^ y^−1^. In comparison, fifty years after rewetting the naturally regenerated unmanaged peatland forest show decreased C loss, which is due to lower peat mineralization and C accumulation in biomass. In 160 years they achieve C dynamics similar to the pristine bog forest—with a moderate C sequestration rate of 0.1 t C ha^−1^ y^−1^. Restoring a peat extraction site to bog ecosystem would become C neutral in nearly 20 years, and onwards continuous mean annual C sequestration of 0.22 t C ha^−1^ y^−1^ is assumed (Supplementary Table 1).

Peatland forest drainage can give a significant increase in short-term C capture in biomass (Lohila et al. 2011), however, long-term dynamics in C stock and peat mineralisation remain largely unknown.

Restoration of crop plantations established on tropical peatlands and other dramatically altered peatlands differs from restoration of temperate and boreal bogs because the source community is predominantly swamp forest where peat-forming material is predominantly wood. Above ground and below ground litter formation under the reforestation and management with moderate drainage can more easily compensate peat mineralization than in temperate and boreal areas (Couwenberg et al. 2010).

## Conclusions

Understanding the dynamics of GHG fluxes caused by land-use change is essential for successful peatland restoration. Our analysis identified several contradictory research results and gaps in a deep understanding of these processes. Notably, there is a lack of GHG flux data for most of tropical drained and restored peatlands, with the exception of oil palm plantations. Another important issue is retention of C in restored peatlands, where we can differentiate between short-term C capture (GHG exchange between the peatland and atmosphere) and long-term C capture (accumulation in soil). Based on our analysis of literature sources and own research results from Estonia, rewetting of former peat extraction areas for further management or conservation is the only viable approach for long-term C sequestration. In contrast, afforestation combined with continuous peatland drainage may have short-term economic benefits but leads to C losses in the long term.

Uncertainties in long-term estimations of C storage and GHG flux dynamics are remarkably high and do not allow for exact predictions. However, even educated guesses can be valuable for decision making on further management of peatlands. To make accurate estimations, it is crucial to investigate the full combined impact of hydroclimate change, microbial processes, and vegetation on GHG emissions from restored peatlands.

### Supplementary Information

Below is the link to the electronic supplementary material.

Supplementary file1 (DOCX 16 kb)

## Data Availability

Data are available within the article and its supplementary materials. For additional data or questions, please contact the authors.
