# Supplementary material for: Peatland restoration pathways to mitigate greenhouse gas emissions and retain peat carbon
Source: Biogeochemistry. 2023 Dec 8;167(4):523–43. doi: 10.1007/s10533-023-01103-1 (PMC11068583; doi:10.1007/s10533-023-01103-1)
Supplement: Supplementary file 1 — Supplementary file1 (DOCX 16 kb) [file 10533_2023_1103_MOESM1_ESM.docx]

**Supplementary Table 1**: Input parameters and assumptions for the conceptual model of long-term carbon stock dynamics in peatland ecosystems (Fig. 3).

|  | **Land use class** | | | | | |
| --- | --- | --- | --- | --- | --- | --- |
|  | Natural | Natural | Managed forest | Former milled peat production field | Former milled peat production field | Former milled peat production field |
|  | Open or treed ombrotrophic bog | Ombrotrophic bog forest | Drained ombrotrophic bog | Rewetted bog | Natural revegetation, no management | Afforestation, managed |
| **C stock in peat layer** | |  |  |  |  |  |
| **Initial soil carbon stock (year-0), C (t/ha)** | 686 | 686 | 686 | 686 | 686 | 686 |
| Depth of initial peat (m) | 2 | 2 | 2 | 2 | 2 | 2 |
| Average bulk density of peat profile (BD dry; g/cm3) | 0.07 | 0.07 | 0.07 | 0.07 | 0.07 | 0.07 |
| Average peat layer C content (%) | 49 | 49 | 49 | 48 | 48 | 48 |
| **Final soil carbon stock (year 300), C (t/ha)** | 752 | 716 | 408 | 733 | 365 | 243 |
| **Soil C stock change (t/ha)** | 66 | 30 | -278 | 61 | -307 | -429 |
| **C stock in wooded biomass (t/ha)** |  |  |  |  |  |  |
| **Initial wood biomass C stock (year-0), C (t/ha)** | 3 | 100 | 0 | 0 | 0 | 0 |
| *Annual increment m^3^/yr* | *0* | 0.1 | 4 | 0.2 | 0 ... 4 | 0 ... 6 |
| Annual increment of above-ground wood biomass, C t/ha | 0 | 0.028 | **1.060** | 0.053 | 0 ... 1.060 | 0 ... 1.591 |
| Annual increment of below-ground wood biomass, C t/ha | 0 | 0.023 | **0.157** | 0.008 | 0 ... 0.157 | 0 ... 0.236 |
| Carbon removal by management C (t/ha) | 0 | 0 | **155.886** | 0 | 0 | 467.657 |
| Harvesting period (years) | 0 | 0 | 100 | 0 | 0 | 100 |
| **Final wood biomass C stock (year-300), C (t/ha)** | 3 | 115.353 | 209.432 | 16.744 | 114.248 | 80.320 |
| **Wooded biomass C-stock change (t/ha)** | 0 | 15.353 | 209.432 | 16.744 | 114.248 | 80.320 |
| **Total C-stock change 300 a jooksul, C t/ha** | 66 | 45 | -69 | 78 | -193 | -349 |
| **Ecosystem C stock after 300 yr** | 752 | 731 | 617 | 750 | 479 | 323 |
| NEE (net ecosystem exchange), C t/ha*a | -0.22 | -0.1 | 1.00 | 3.7 ... -0.22  *(dynamic)* | 3.7 ... -0.1  *(dynamic)* | 3.7 ... 1.0  *(dynamic)* |

**Calculation:**

**Cstock in year n = *f* (C_n-1_– NEE_n_ + WB_n_- H_n_)**

C –ecosystem carbon stock

n –year

WB – wood biomass carbon (aboveground + belowground)

H – harvesting (equal to zero if no harvest or thinning of the stand)

**Data sources:**

Average bulk density of peat profile (BD dry; g/cm3), average peat layer C content (%): Kull, 2016

Annual increment m^3^/yr: Estonian Forestry Register (<https://register.metsad.ee/#/>)

Ratio between above and below-ground woody biomass, and C content in wood: Külla 1997; Kask & Pikk 2009; Lutter et al. 2019

NEE: Wilson et al. 2016; Aro et al. 2022

**References:**

Aro L, Assmuth A, Haltia E, Hellsten S, Larmola T, Lempinen H, Lindfors L, Lohila AK, Lång K, Miettinen A, Minkkinen K, Myllys M, Nieminen M, Ollikainen M, Ojanen P, Sarkkola S, Sorvali J, Seppälä J, Tolvanen A, Vainio A, Wall A, Vesala T (2022) Alternatives for the use of peatlands in carbon-neutral Finland. The Finnish Climate Change Panel Report No 2/2022. In Finnish). <https://doi.org/10.31885/9789527457115>

Kask R, Pikk J (2009) Second thinning Scots pine wood properties in different forest site types in Estonia. Baltic Forestry 15(1): 97-104.

Kull A (2016) Buffer zones to limit and mitigate harmful effects of long-term antropogenic influence to maintain ecological functionality of bogs, stage II. Estonian Environmental Investment Centre (KIK), 183 p. (In Estonian).

<https://4ce0b57b-a630-4e1d-8a88-d1e1a7a51b96.filesusr.com/ugd/6b6658_446958f4118b44a2a68812820c31119b.pdf>

Külla T (1997) Below-ground and above-ground structure of a middle-aged Scots Pine stand and Norway Spruce stands. MSc Thesis, Estonian University of Life Sciences, Tartu, 100 p. (In Estonian).

Lutter, R.; Kõlli, R.; Tullus, A.; Tullus, H (2019). Ecosystem carbon stocks of Estonian pre-mature and mature managed forests: effects of site conditions and overstorey tree species. Eur J Forest Res 138: 125−142. <https://doi.org/10.1007/s10342-018-1158-4>

Wilson D, Blain D, Couwenberg J, Evans CD, Murdiyarso D, Page SE, Renou-Wilson F, Rieley JO, Sirin A, Strack M, Tuittila ES (2016a) Greenhouse gas emission factors associated with rewetting of organic soils. Mires Peat 17:1–28. <https://doi.org/10.19189/MaP.2016.OMB.222>
